# Comparison of Effectiveness of Hiora SG Gel With Triamcinolone Acetonide Gel in Recurrent Aphthous Stomatitis

**DOI:** 10.7759/cureus.40403

**Published:** 2023-06-14

**Authors:** Nirmal G Nayak, Panthi Modi, Swasti Shah, Pranjal Patel, Devarshi Devangkumar Patel, Riddhi Rohitbhai Patel, Devanshi Hapani

**Affiliations:** 1 Public Health Dentistry, Karnavati School of Dentistry, Gandhinagar, IND; 2 Dentistry, AMC Dental College & Hospital, Ahmedabad, IND; 3 Dentistry, Sure Align Orthodontix n Dentistry Clinic, Ahmedabad, IND; 4 Dentistry, Smile and Face Dental Care & Implant Centre, Ahmedabad, IND

**Keywords:** severity score, triamcinolone, hiora sg gel, polyherbal formulation, stomatitis, aphthous

## Abstract

Background

The oral condition known as recurrent aphthous ulceration (RAU) or recurrent aphthous stomatitis (RAS) is very prevalent. Its etiopathogenesis is unknown; hence, symptomatic therapy is all that can be offered if it manifests clinically. Lesion care aims to minimize discomfort and the frequency of relapses by bringing active illness under local control in the affected area. The current treatment options that may have negative side effects include the use of topical and systemic steroids, antibiotics, cauterization, and laser therapy.

Objectives and Importance

This study aimed to compare the efficiency of HiOra SG gel with triamcinolone acetonide gel in the management of RAS.

Materials and Methods

Fifty individuals with RAS were recruited for the trial and randomly assigned to either group I (HiOra SG gel) or group II (0.1% triamcinolone acetonide ointment; Oraways). After each meal for a total of 10 days, those with mouth ulcers were instructed to topically administer the drugs. The clinical data were analyzed by comparing the ulcer severity scores from the first, fifth, and 10th days.

Results

There was a statistically significant (p = 0.001) reduction in reported pain, pain duration, and overall ulcer severity across all groups. After therapy, however, neither the HiOra gel group nor the triamcinolone group showed any discernible improvement over the other.

Conclusion

The present study’s findings corroborate the efficacy of HiOra SG gel in the treatment of RAS when compared to triamcinolone acetonide gel (0.1%). In this trial, no patients had any negative reactions to HiOra SG gel. In the future, further studies are needed with larger samples to prove its benefits.

## Introduction

The condition known as recurrent aphthous ulcer (RAU) or recurrent aphthous stomatitis (RAS) is one of the most common oral illnesses seen in the general population [1]. Stomatitis is described as an inflammation of the oral depression brought on by mechanical, material, thermal, bacterial, viral, or radiation injury or as a secondary manifestation of any underlying sickness [2]. RAS may have several causes, including environmental factors, genetics, malnutrition, food, hormonal changes, and immune system diseases [2]. In fundamental circumstances, such as Behcet’s disease, Reiter’s disease, Sweet’s disease, Crohn’s disease, Celiac disease, cyclic neutropenia, and so on, it may also be presented as widespread ulcerations [2,3]. Quitting smoking, oral trauma, and food allergies are additional risk factors for aphthous stomatitis [2]. Patients with RAS often do not present with any other systemic diseases or health issues.

Aphthous stomatitis that keeps recurring may be treated with a variety of methods, including silver nitrate cauterization; antibiotics; laser therapy; topical anesthetic preparations including benzocaine, dyclonine hydrochloride, and lidocaine with diphenhydramine; and systemic and topical corticosteroids. Two of the potential side effects of using topical corticosteroids include oral candidiasis and cutaneous atrophy [4,5]. This research compared the topical administration of a polyherbal formulation (HiOra SG gel) to triamcinolone acetonide 1% (Oraways) for the treatment of RAS, since the use of herbal medications is becoming more popular due to their lower side effects.

Guava leaves, Indian cherry leaves, licorice, turmeric, pomegranate flowers, betel leaves, aloe vera, capsicum, and noni fruit extracts have been used for mouth ulcers. The HiOra SG gel is formulated with a blend of herbs, including *Glycyrrhiza glabra*, *Jasminum grandiflorum*, *Azadirachta indica*, *Ocimum basilicum*, *Boerhavia diffusa*, *Syzygium aromaticum*, and Triphala. Although natural remedies for RAS have been shown to be effective in a few studies [6-8], more research is needed to confirm its benefits. HiOra SG Gel is a herbal gel that contains various natural ingredients, such as Yashtimadhu (licorice) and Khadira (*Acacia catechu*). These ingredients have antimicrobial, anti-inflammatory, and wound-healing properties. When applied topically to the affected area, HiOra SG Gel helps reduce inflammation, relieve pain, and promote the healing of mouth ulcers [6]. Triamcinolone acetonide is a corticosteroid medication that belongs to a class of drugs called glucocorticoids. It has potent anti-inflammatory and immunosuppressive properties. When applied topically as a gel, it helps to reduce inflammation, swelling, redness, and pain associated with mouth ulcers. Triamcinolone acetonide gel works by suppressing the immune response and reducing the release of inflammatory substances in the affected area [7].

The purpose of this investigation was to compare the performance of triamcinolone acetonide 1% gel for RAS with that of a polyherbal formulation (HiOra SG gel).

## Materials and methods

This clinical study compared the effectiveness of HiOra SG gel to that of triamcinolone acetonide gel in the treatment of RAS. Ethical approval was obtained prior to conducting the research with an institutional review board number KSD/IEC/2022/12. Fifty people who were seen in the outpatient portion of the Department of Public Health Dentistry, Karnavati School of Dentistry, Gandhinagar, India, participated in the research. The research included two groups, and the sample size was calculated based on sample size calculation at a 95% interval and population affected and number of people affected by ulcers, which were calculated as 20 participants in each group. Accordingly, in this study, 25 participants per group were taken into consideration from the department. In group I, 25 participants were treated with HiOra SG gel; in group II, 25 participants were treated with triamcinolone acetonide. All participants were given an explanation of the study, and they gave their informed permission in writing. Anyone over the age of 18 who was diagnosed with chronic aphthous stomatitis minor ulcers is eligible to participate. Individuals with allergies to pharmaceuticals used topically, women who were pregnant or breastfeeding, or individuals who had any other systemic illness or chronic ulcerative condition, or those who used any long-term drugs were not eligible. Subjects were placed in groups by a convenient sampling method. 

Aphthous ulcers that were smaller than 1 cm in diameter were considered minor RAS ulcers. Patients who met the study’s diagnostic and eligibility criteria were enrolled.

In the patient proforma, we record the patient’s vital parameters and their medical and dental histories in great detail. The first observer documented the patient’s clinical observations on the first, fifth, and 10th days, following evaluation in a specific proforma for this research. In order to achieve double blinding, the drugs were packaged similarly and administered by a second observer. Drug A was assigned to HiOra SG gel, whereas drug B was assigned to triamcinolone acetonide gel. The first observer and investigator were given the drug information after the trial was complete. People with mouth ulcers were instructed to apply either HiOra SG gel or 0.1% triamcinolone acetonide gel to the affected area four to five times daily after meals for 10 days. Clinical data were entered in patient proforma and scored using the ulcer severity index. Neither external nor internal medications were used at any point during the study. At the same time, local skin examinations were performed at each visit. Participants were free to drop out of the study whenever they saw fit. The ulcer severity score (USS) was developed by Tappuni et al. [9]. Ulcers were evaluated for severity on days five and 10; ulcer count, ulcer location, ulcer size, and ulcer pain are the four variables that were combined to determine the severity of ulcers. During each follow-up, data on how long the pain lasted were also recorded.

SPSS, version 16.0 (SPSS Inc., Chicago, IL) was used for the statistical analysis. For quantitative variables with a normal distribution, data were presented as mean ± standard deviation (SD). The formula n = t^2^ × p(1 − p) was used to determine the appropriate size of the sample to collect. The student’s t-test is used to compare quantitative variables, and the analysis of variance (ANOVA) with a post hoc test is used to determine statistical significance. The chi-square test was used to examine the differences in gender distribution across the groups. In this study, a p-value of less than 0.05 was used as the threshold for statistical significance.

## Results

Four (16%) were under the age of 20, 13 (52%) were between the ages of 21 and 30, six (24%) were between the ages of 31 and 40, two (8%) were between the ages of 41 and 50, and none were present between the ages of 51 and 60. In group II, four (16%) were less than 20 years old, 14 (56%) were between 21 and 30 years old, five (20%) were in their 31s to 40s, one (4%) was in their 41s to 50s, and one (4%) was in their 60s. Toward the current investigation, patients with RAS tended to cluster toward the younger end of the study age range (21-30 years old) for both groups. There were six boys (24% of group I) and 19 girls (76% of group I). In group II, there were nine males (36% of the total) and 16 females (64%). There were more women than men diagnosed with RAS when comparing the two groups. For day one, the p-value was 0.856; for day five, the p-value was 0.765; and for day 10, the p-value was 0.351. In this research, comparing pain assessments across the two groups did not provide statistically significant results (p > 0.05). This demonstrated that both groups were similarly effective in relieving pain resulting from RAS. Pain ratings on days one, five, and 10 of the study are compared between the two groups in Table 1.

**Table 1:** Statistical analysis of pain levels in the two groups

					95% confidence interval for mean		
		Mean	Standard deviation	Standard error	Lower bound	Upper bound	p-value
Day 1	HiOra	5.45	1.652	0.22	5.81	7.41	
	Triamcinolone acetonide (0.1%)	5.40	1.897	0.274	5.75	7.48	0.856
Day 5	HiOra	3.68	1.871	0.275	2.01	3.7	
	Triamcinolone acetonide (0.1%)	3.6	1.575	0.2	2.05	3.3	0.765
Day 10	HiOra	0.47	1.02	0.15	0.11	0.80	
	Triamcinolone acetonide (0.1%)	0.27	0.854	0.157	0.01	0.65	0.351

Table 2 displays a comparison of the total USSs on days one, five, and 10 between the two research groups.

**Table 2:** Scores for the overall severity of ulcers compared across the study groups

		Mean	Standard deviation	Standard error	Lower bound	Upper bound	p-value
Day 1	HiOra	14.01	9.610	2.116	11.15	15.16	
	Triamcinolone acetonide (0.1%)	12.11	7.145	0.876	11.10	14.81	0.415
Day 5	HiOra	8.19	8.179	1.724	5.54	11.14	
	Triamcinolone acetonide (0.1%)	5.01	5.461	0.775	5.28	7.10	0.356
Day 10	HiOra	2.15	3.107	0.610	1.20	2.56	
	Triamcinolone acetonide (0.1%)	1.75	4.165	0.651	0.78	2.40	0.519

The p-values were 0.415 for day one, 0.356 for day five, and 0.519 for day 10. The review groups did not significantly differ in USSs (p > 0.05). After therapy, there was no discernible difference in USSs between the two groups.

On days five and 10, we compared the length of time patients in the HiOra group had discomfort to those in the triamcinolone group. As the p-values for day five after using HiOra gel and day 10 after using triamcinolone gel were both >0.05, it was determined that there was no statistically significant difference in the pain duration between the two treatments. Accordingly, the results indicated that neither group experienced significantly longer periods of pain than the other (Table 3).

**Table 3:** HiOra and triamcinolone acetonide groups compared based on how long their discomfort lasted

		Mean	Standard deviation	Standard error	Lower bound	Upper bound	p-value
Day 5	HiOra	3.15	0.750	0.145	3.10	4.15	
	Triamcinolone acetonide (0.1%)	3.10	0.871	0.161	2.75	3.65	0.455
Day 10	HiOra	1.85	1.501	0.312	0.75	1.10	
	Triamcinolone acetonide (0.1%)	1.89	1.525	0.315	0.55	1.84	0.410

## Discussion

Many people are unable to tolerate the side effects of the currently available treatment options for RAS. Because of this, achieving the management objectives for this illness calls for an innovative therapeutic strategy [1]. For this reason, we conducted a comparison of the effectiveness of the polyherbal formulation HiOra SG gel (used for the treatment of RAS) with that of triamcinolone acetonide (0.1%) gel. Umamaheswari et al. [10] used *J. grandiflorum* (also known as Jati) extracts in an in vitro investigation, describing how they had antiulcer and antioxidant characteristics. Licorice’s anti-inflammatory, antibacterial, and antifungal properties were reviewed by binti Bismelah et al. in 2016 [11]. Using an incision wound as an example, Yadav et al. [12] report that Triphala possesses antimicrobial and wound-healing action. Having all these qualities is crucial while dealing with RAS. Traditional medicine based on clove, also known as Syzygium aromaticum, dates back to at least 240 BC in Asia. Pain from ulcers may be managed using its analgesic and moderate anesthetic properties, which work by blocking pain signals from reaching the brain. The compound also exhibits antibacterial properties [12].

HiOra SG gel comprises a number of herbal medications that may be used successfully in the therapy of recurrent oral ulcers due to their combination of anti-inflammatory, anti-ulcer, antibacterial, immunomodulatory, and analgesic activities. Tappuni et al. calculated the ulcer severity by looking at six different factors: the number of ulcers, their location, their size, how long they lasted without an ulcer, how painful they were, and how often they occurred [9]. To determine the severity of the illness, an overall USS was calculated [9]. After applying each topical drug, the time till the discomfort subsided was also determined. Research conducted by Patil et al. [13] on the incidence of recurrent oral ulcers in the Indian community indicated that patients with RAS were most common between the ages of 31 and 40. Among both age groups, the present study saw a peak in the proportion of RAS patients between the ages of 21 and 30.

HiOra SG gel was studied by Sukumaran clinically for the relief of aphthous stomatitis [14]. In this research, pain was shown to lessen with time, with the average pain score dropping from the first to the third week. Pain levels were significantly lower in the HiOra gel group after the second visit compared to the 5% amlexanox group, according to research by Bell [15]. This finding ran counter to the direction we are taking in this study.

The pain was significantly reduced in the HiOra gel group compared to the placebo group after the first, second, and third visits (p = 0.001). Pain intensity ratings were compared between the HiOra gel group and the triamcinolone acetonide group during the course of this trial, with p-values of 0.856, 0.765, and 0.351 on days one, five, and 10, respectively. According to the results of this study, neither group significantly outperformed the other in terms of their ability to reduce pain.

After therapy with topical corticosteroids, the USS considerably reduced from 34.6 to 27.4 with a p-value of 0.001. This was determined by a clinical evaluation of RAS performed by Tappuni et al. [9]. As the p-values on days one, five, and 10 for the comparison between the USSs in the HiOra gel group and the triamcinolone acetonide group were 0.415, 0.356, and 0.519, respectively, no statistically significant difference was found. Bell [15] found that neither the HiOra gel group nor the amlexanox group significantly reduced the number of ulcers on either the first or second follow-up visits. The average size of ulcers was significantly less in the amlexanox group than in the HiOra gel group, indicating a positive effect.

Using HiOra SG gel and triamcinolone acetonide gel, the current research measured how long the discomfort lasted following the intervention. Both the five-day and 10-day pain durations were not significantly different between the HiOra gel and triamcinolone acetonide groups. This means that both groups benefited equally from RAS’s ability to lessen their discomfort for a longer period of time. According to the results of the current research, a polyherbal composition called HiOra SG gel is useful for treating RAUs. So herbal treatments and triamcinolone acetonide with their anti-inflammatory properties can just be considered symptomatic remedies that lessen pain and discomfort. As a result, you won’t have to cope with the drawback of traditional therapies like topical corticosteroids. To verify HiOra SG gel’s effectiveness, similar research with a larger sample size may be conducted. The USS described here may be adapted for use in everyday dentistry practice, to both evaluate the severity of illness and demonstrate the effectiveness of different medications for treating RAS [16].

There are limitations that include less sample size and only two treatment modalities compared. CONSORT guidelines could be used in future studies so that the proper protocol is set up. However, future studies may use the USS and a larger sample size to confirm HiOra SG gel’s efficacy.

## Conclusions

In terms of relieving ulcer severity, pain duration, and pain intensity, the findings show that there was no discernible difference between HiOra SG gel and triamcinolone acetonide gel. In this trial, no patients had any negative reactions to HiOra SG gel. Therefore, the present study’s findings suggest that this polyherbal formulation may be a viable alternative to the widespread use of topical corticosteroids in the treatment of this condition.
